# Supplementary material for: Auxin-independent effects of apical dominance induce changes in phytohormones correlated with bud outgrowth
Source: Plant Physiol. 2023 Jan 24;192(2):1420–34. doi: 10.1093/plphys/kiad034 (PMC10231355; doi:10.1093/plphys/kiad034)
Supplement: kiad034_Supplementary_Data [file kiad034_supplementary_data.pdf]

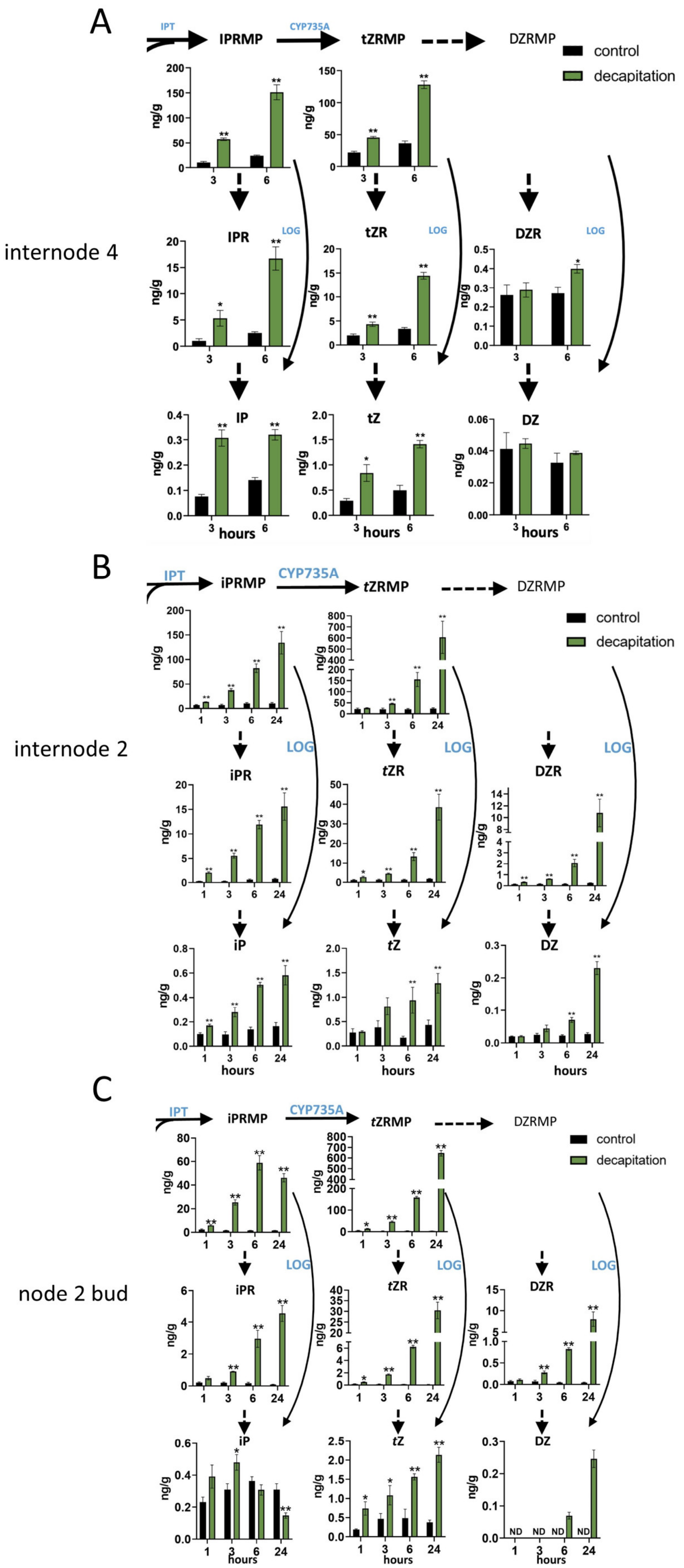
A
internode 4
B
internode 2
C
node 2 bud
IPT
CYP735A
LOG
iPRMP
tZRMP
DZRMP
iPR
tZR
DZR
iP
tZ
DZ
control
decapitation
ng/g
hours
ND

Supplemental Figure S1. Endogenous CK level changes in internodes and node 2 after decapitation. CK level changes in Internode 4 (A), internode 2 (B), node 2 bud (C) at 1, 3, 6, and 24 h after decapitation. Node 2 was about 12 cm from the decapitation site. * *P*<0.05, ***P*< 0.01, Student's *t* test, n = 4. Each replicate contains 20 individual buds. Values are mean ± SE. Abbreviations: iPRMP, isopentenyladenosine-5'-monophosphate; tZRMP, trans-zeatin riboside-5'-monophosphate; DZRMP, dihydrozeatin riboside-5'-monophosphate; iPR, isopentenyladenosine; tZR, trans-zeatin riboside; DZR, dihydrozeatin ribodide; iP, isopentenyladenine; tZ, trans-zeatin; DZ, dihydrozeatin; IPT, adenosine phosphate-isopentenyltransferase; LOG, cytokinin phosphoribohydrolase 'Lonely guy'; CYP735A, cytochrome P450 mono-oxygenases. ND, not detected.

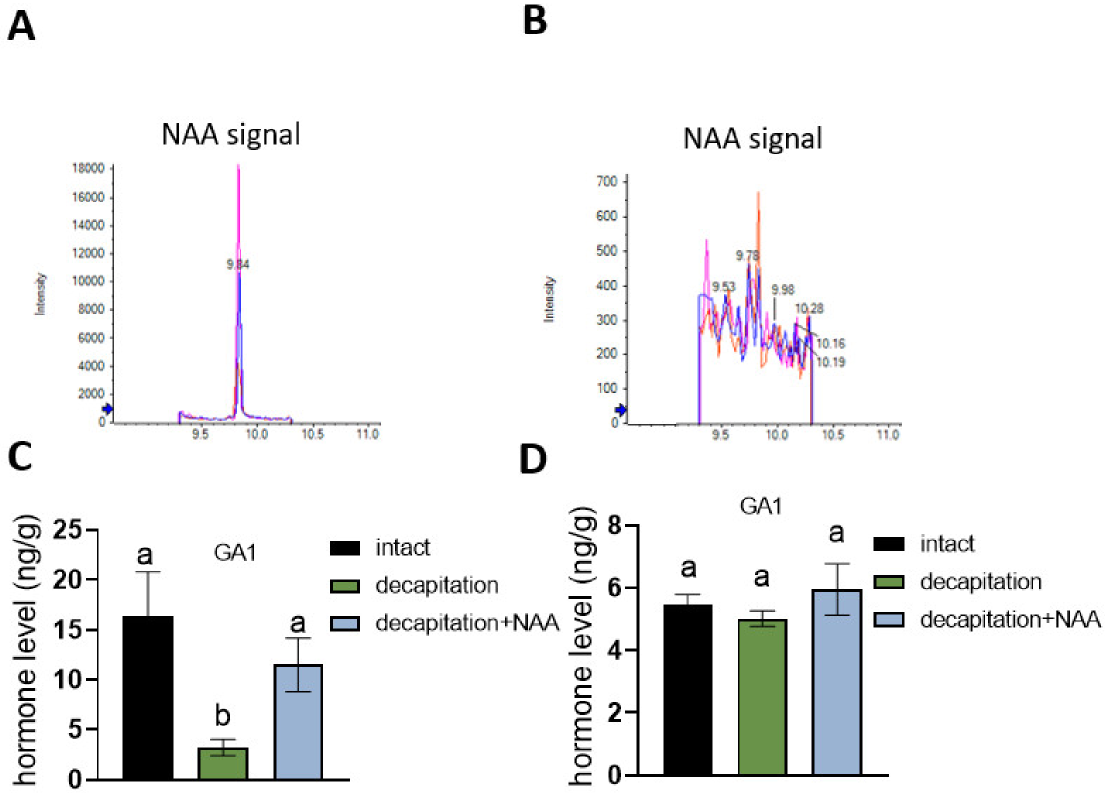

Supplemental Figure S2. NAA and GA level changes after decapitation and treatment with 3g/L NAA applied to the decapitated stump above internode 4. (A, B) Extracted ion chromatograms showing a detectable NAA signal (186.9-›141 m/z transition, 9.8 min retention time) in internode 4 (A) and undetectable NAA signal in internode 2 (B) 4 h after decapitation and treatment with 3g/L NAA applied to the decapitated stump above internode 4. (C, D) Endogenous GA_1 levels in internode 4 (C) and internode 2 (D) 4 h after decapitation of decapitated plants treated either with mock or 3 g/L NAA above internode 4. Values are mean ± SE, *n* = 4. Multiple comparison tests were performed with one-way ANOVA; *n* = 4.

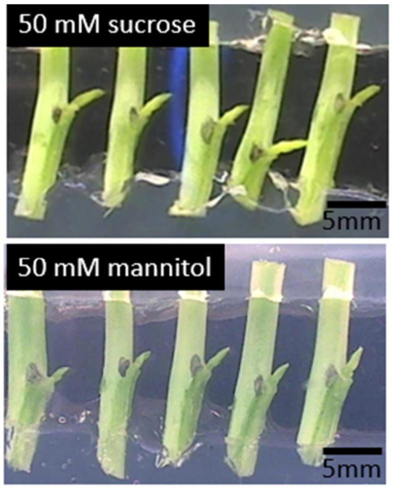

Supplemental Figure S3. Buds of nodal stem segments exhibit growth after 24 h treatment with 50 mM sucrose compared to 50 mM mannitol control.

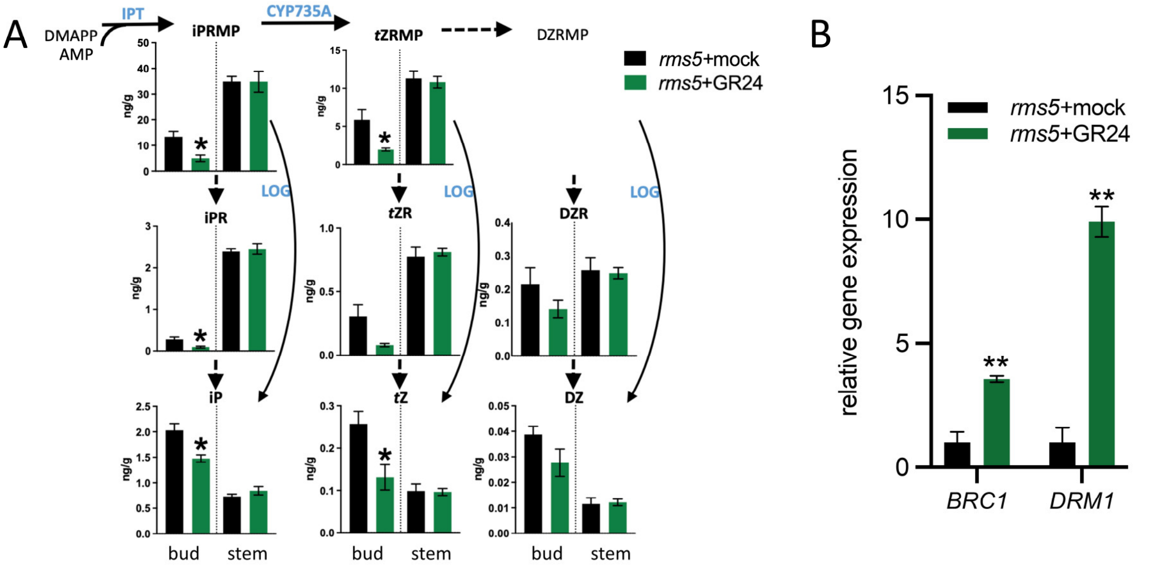

Supplemental Figure S4. Endogenous CK level and bud dormancy gene changes in *rms5* node 2 buds and internode 2 treated with or without 10 μM of GR24. (A) Endogenous CK levels in *rms5* node 2 buds and internode 2 treated with or without 10 μM of GR24 on node 2 buds for 6 h. n = 3. (B) Expression of bud dormancy genes in *rms5* node 2 buds treated with or without 10 μM of GR24 for 6 h. *n* = 3. Values are mean ± SE. Each replicate contains 20 individual buds. * *P*<0.05, ***P*< 0.01, Student's t test.

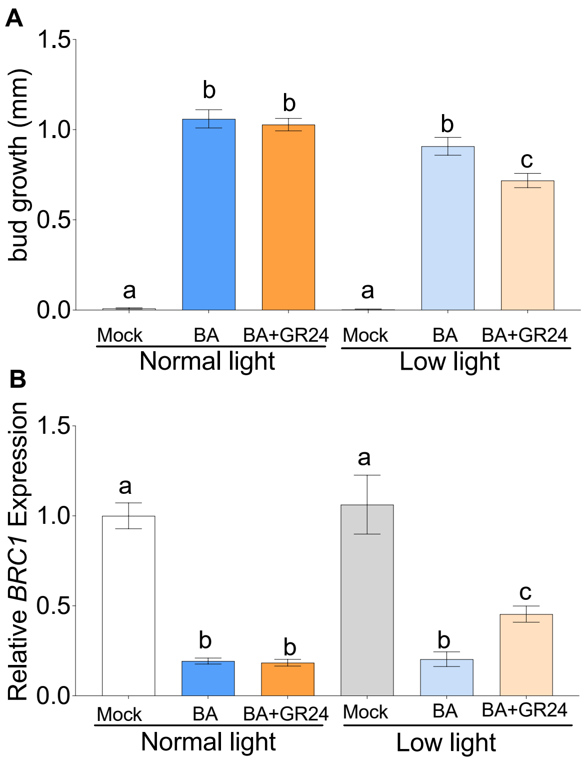

Supplemental Figure S5. The early response of CK treated buds to SL is reduced under higher light. Effect of GR24 on BA-induced bud outgrowth at 24 h (A) and *BRC1* expression at 6 h (B) after treatment. Treatments were 50 μM BA ± 5 μM GR24; normal light, 150-200 μmol $m^{-2}s^{-1}$; low light, 50-75 μmol $m^{-2}s^{-1}$. Expression of *BRC1* in the bud at node 2 is represented relative to the high light mock control. Different letters on the top of columns indicate significant difference with one-way ANOVA. Values are mean ± SE; *n* = 6 plants (A) or 6 pools of 6 plants (B).

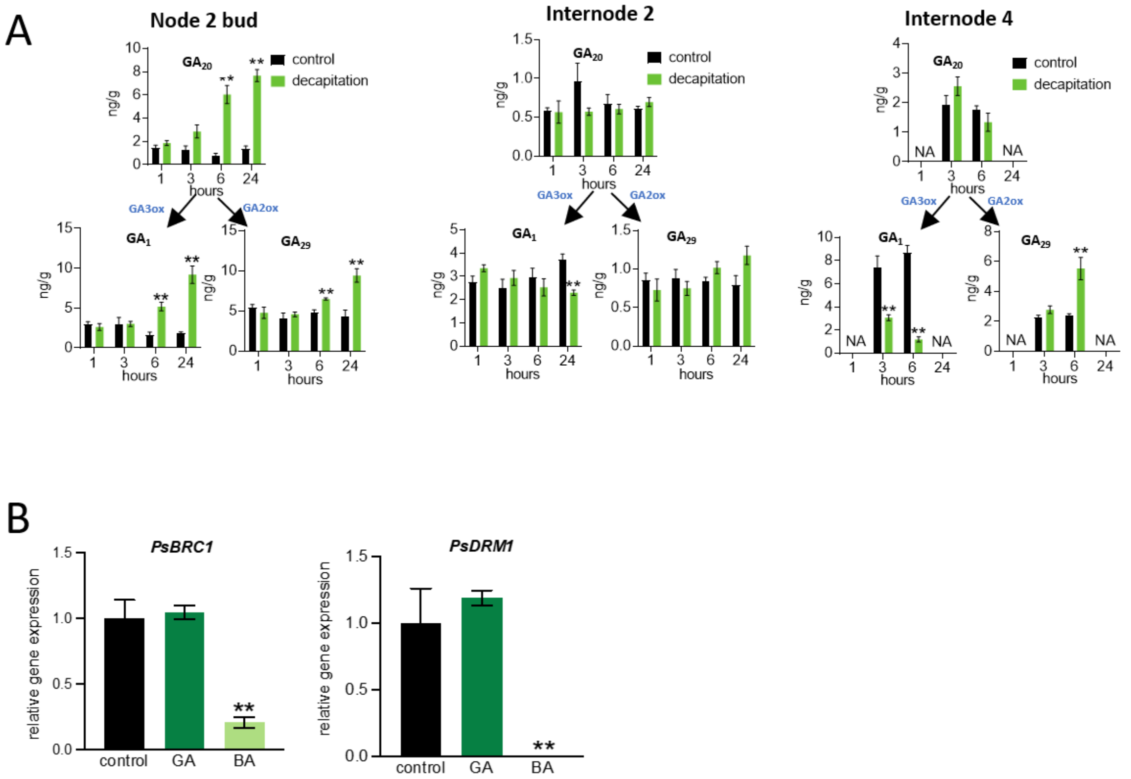

Supplemental Figure S6. Endogenous GA level changes after decapitation and bud dormancy gene changes in response to exogenous GA and CK treatments. (A) Changes in levels of endogenous GAs (GA_1, GA_{20}, GA_{29}) in node 2 bud, internode 2 and internode 4 after decapitation. Each replicate contains 20 individual buds; n = 4. Values are mean ± SE. * P <0.05; ** P < 0.01, with respect to the directly comparable treatment; Student's t test. NA, not available. (B) Node 2 axillary buds were treated with 2.9 mM GA or 50 µM BA for 6 hours. Values are mean ± SE, n = 4. Each replicate contains 20 individual buds. **P < 0.01 compared to mock control, Student's t test.

Supplemental table S1. Gene accession numbers and primers used in the study.

gene name	accession number	forward primer	reverse primer
PsEF1α	PsCam044181	TGTGCCAGTGGGACGTGTTG	CTCGTGGTGCATCTCAACGG
PsGADPH	PsCam050033	TCGGACTTCAGGGATGTGTATT	GCTGGTGCGGAGTTTATCTG
PsTUB2	PsCam044217	AGATGGCTTCAACTTTCATTGG	GCTCTCGGCTTCGGTGA
PsBRC1	PsCam007042	CCGACGCCACCAACAATATC	TCTTTGCAACTTCCAGCGAC
PsDRM1	PsCam059622	TATTGTTGCTGGCCCTCAACCG	TATTGTTGCTGGCCCTCAACCG
PsIPT1	PsCam038526	ACCGTCTTGATGCTACGGAGGTTGTGC	TCTAATGGGTTACCCCTGCCA
PsIPT2	PsCam038920	TGGCAGCAACATCATCCTCTGCCTGC	ACCTGTGGCCCCCATTATCACTAC
PsLOG1	PsCam016984	GGAGGCAGCATTGGTTTGAT	TTGATGCATGTCAGCCACTG
PsLOG2	PsCam052396	CTTGGGCTCAACTTGGCATC	TGCAACTGTCCATTTTCCCAG
PsLOG3	PsCam039893	GGAGGAGGAAGCATTGGACT	AGCCATCTCAGCCTTCCTTT
PsLOG4	PsCam039655	ATGCCTTCATTGCTCTCCCT	CCTTCATCAACGGCCTTGTC
PsLOG5	PsCam030985	GTGGGAGAAGTGAGAGCTGT	GGGCAGCTGGTGTTACAAAA
PsLOG6	PsCam026437	GTGGTGGTGGTGAATACGTG	TCCCAGGACTACTACCACAAA
PsLOG7	PsCam031214	TTCCCAAGACACTCATGCCT	CCGGCTTGTCATGAATTCCA
PsCKX1	PsCam034360	ACTCTCTCGAACGCTGGAAT	ATTGACCAAGCCCTCCAAGA
PsCKX3	PsCam000181	AAACGACCCAAAAGCCCTTG	CCTGTCCTCTTGCCGCTATA
PsCKX5	PsCam034398	AAATGTATGTTGATGTTTGGGGTGG	TTTGAGGACCGTGATTAAAGGTTTG
PsCKX7	PsCam042803	TAGCATTGCTTCGATTTATTCCACC	CATCCAATTCTCGTGTGACAAGTAG
PsARR5	PsCam044483	GGTGAAGGAAAGAAAGTGGAAGG	TGAGGACGATAGAGATGAAATGGA
